# Ketogenic Diet: A Nutritional Therapeutic Tool for Lipedema?

**DOI:** 10.1007/s13679-023-00536-x

**Published:** 2023-11-04

**Authors:** Ludovica Verde, Elisabetta Camajani, Giuseppe Annunziata, Antoanstefan Sojat, Ljiljana V. Marina, Annamaria Colao, Massimiliano Caprio, Giovanna Muscogiuri, Luigi Barrea

**Affiliations:** 1 Department of Public Health, University of Naples Federico II, Via Sergio Pansini 5, 80131 Naples, Italy; 2 Centro Italiano per la cura e il Benessere del Paziente con Obesità (C.I.B.O), Unità di Endocrinologia, Diabetologia e Andrologia, Dipartimento di Medicina Clinica e Chirurgia, Università degli Studi di Napoli Federico II, Via Sergio Pansini 5, 80131 Naples, Italy; 3 Department of Human Sciences and Promotion of the Quality of Life, San Raffaele Roma Open University, 00166 Rome, Italy; 4 Laboratory of Cardiovascular Endocrinology, IRCCS San Raffaele, Rome, Italy; 5 Department of Experimental Medicine, Luigi Vanvitelli University of Campania, Naples, Italy; 6 Department for Obesity, Metabolic and Reproductive Disorders, Clinic for Endocrinology, Diabetes and Metabolic Diseases, University Clinical Centre of Serbia, Belgrade, Serbia; 7 Dipartimento di Medicina Clinica e Chirurgia, Diabetologia ed Andrologia, Unità di Endocrinologia, Università Federico II, Via Sergio Pansini 5, 80131 Naples, Italy; 8 Cattedra Unesco “Educazione Alla Salute E Allo Sviluppo Sostenibile”, University Federico II, Naples, Italy; 9 Dipartimento di Scienze Umanistiche, Università Telematica Pegaso, Centro Direzionale, Via Porzio, Isola F2, 80143 Naples, Italy

**Keywords:** Lipedema, Inflammation, Obesity, Very low-calorie ketogenic diet, Diet, Nutrition, Fat

## Abstract

**Purpose of Review:**

This review aims to provide an overview of the current evidence on the efficacy, also considering the anti-inflammatory properties and safety of very low-calorie ketogenic diet (VLCKD) as a potential treatment for lipedema, particularly in the context of obesity.

**Recent Findings:**

Lipedema is a chronic disease characterized by abnormal and painful fat buildup on the legs and/or arms. It is often misdiagnosed as obesity or lymphedema. However, although lipedema and obesity can coexist, unlike obesity, lipedema usually affects the legs and thighs without affecting the feet or hands, and the abnormal deposition of adipose tissue in lipedema is painful. The current lifestyle interventions are often unsuccessful in the management of lipedema. There is no consensus on the most effective nutritional approach for managing lipedema. Recent studies have suggested that VLCKD may be an effective treatment for lipedema, demonstrating that it is also superior to other nutritional approaches such as Mediterranean diet or intermittent fasting.

**Summary:**

Lipedema is a chronic and debilitating disease characterized by abnormal and painful accumulation of adipose tissue in the legs. VLCKD has been shown to be an effective treatment for lipedema, especially in the context of obesity, due to its anti-inflammatory properties. However, further research is needed to determine the long-term safety and efficacy of VLCKD as a treatment for lipedema.

## Introduction

Lipedema is characterized by an abnormal distribution of fat, primarily affecting the lower extremities, upper arms, hips, buttocks, and thighs while sparing the trunk and feet [1]. Symptoms include pain, easy bruising, firm subcutaneous nodules of adipose tissue, and resistance to traditional diet and exercise [2].

The estimated prevalence of lipedema is approximately 1 in 72,000 individuals within the population [3]. However, due to frequent misdiagnosis or underdiagnosis, these numbers are likely to underestimate the actual occurrence of lipedema. While lipedema mainly affects females [4], there have been rare cases reported in males [5–7].

Lipedema is not edema but rather a genetic disorder affecting adipose tissue mass and distribution [8]. It is typically inherited either through X-linked dominant or autosomal dominant patterns. In rare cases, gene mutations like POU1F1/PIT-1 have been observed in short mothers, but their offspring did not exhibit noticeable phenotypic features [8]. Lipedema has also been associated with Williams syndrome, a condition caused by a specific chromosomal microdeletion [9].

Various theories have been proposed to explain the underlying mechanisms of lipedema.

It is speculated that hormonal factors, particularly elevated estrogen levels during puberty or after pregnancy, may play a role in the onset of lipedema in females [4]. Lipedema in males is rare, but few cases have been reported in individuals with liver disease or low testosterone levels [5–7], conditions associated with increased estrogen levels. However, the precise role of estrogens as causative factors in lipedema is not fully understood or firmly established.

Another theory suggests that lipedema involves the loss of elastic tissue and abnormal vasculature [10]. Loose connective tissue comprises structures like blood vessels, lymph nodes, and connective tissue fascia, which contain elastin, a protein that helps maintain their shape. While lymphatic vessels lack elastic fibers, they rely on the surrounding tissue to facilitate the opening and closing of lymph vessels in response to pressure. Similarly, capillaries do not possess elastic tissue themselves, but the loose connective tissue surrounding them does contain elastin. Consequently, as adipose tissue grows in lipedema, the loss of elasticity impairs the ability of lymph vessels to open under increased pressure in the extracellular matrix, leading to capillary leakage in the tissue. This process can result in hypoxia, which stimulates the release of vascular endothelial growth factor (VEGF) and promotes the proliferation of stem cells within the adipose tissue [10].

Distinguishing lipedema from obesity can pose diagnostic challenges. According to the World Health Organization (WHO) guidelines, a body mass index (BMI) exceeding 30 kg/m^2^ is indicative of obesity [11]. Lipedema patients often exhibit an elevated BMI; thus, lipedema is often misdiagnosed as lifestyle-induced obesity [12].

However, it is interesting to note that lipedema can coexist with a state of obesity, which in turn can promote a state of chronic low-grade inflammation [13]. This state of chronic low-grade inflammation in turn can impair lymphatic function, exacerbating adipose tissue accumulation [13]. Thus, lipedema, obesity, and inflammation form an unfavorable vicious cycle.

Considering the wide range of clinical manifestations, according to the severity of lipedema, the treatment of this condition is also not unanimous, and typically used treatments include surgery, compression garments, and physiotherapy [14]. Indeed, adipose tissue accumulation in lipedema is resistant to lifestyle interventions such as diet and exercise [14] and there is currently no consensus on what nutritional approach should be used in its management.

Generally, in order to achieve weight loss, several nutritional strategies and diets are currently available, such as Mediterranean diet [15], intermittent fasting [16], and very low-calorie ketogenic diet (VLCKD) [17]. Of interest, VLCKD has been shown to reduce inflammation more significantly than the others [18]. Current evidence on the efficacy of VLCKD in the context of lipedema is scarce, with only two studies reporting clinical benefits of a ketogenic diet (KD) in subjects with lipedema [19, 20]. Therefore, this review aimed to summarize the current evidence on the efficacy and safety of VLCKD as a potential treatment for lipedema, especially in the context of obesity, highlighting the anti-inflammatory properties of this nutritional approach.

## Definition, Diagnosis, and Treatment

Lipedema is a chronic disease causing a painful bilateral disproportionate swelling of the legs and/or arms, mostly in female subjects, with an onset during or after puberty [21]. It is a condition that is often overlooked and requires a multidisciplinary approach since it can lead to a significant reduction in subjects’ mobility [14]. The prevalence varies from study to study, ranging from as low as 0.1% all the way up to 10% of adult Caucasian female subjects [22, 23].

### Clinical Presentation and Diagnosis

Lipedema has a highly variable progression over time through four stages, leading to disability for several decades if not treated [4]. The fat tissue deposition occurs in arms and legs symmetrically, with a clear demarcation line between hands and feet (the cuff sign), and a clear disproportion between the upper and lower body, waist-to-hip ratio (WHR) and is characterized by heavy, dull pain, exacerbated trough pressure, touch, physical activity, or long periods of sitting [24]. Besides pain, bruising and orthostatic edema are also very common clinical features [1]. Other features include stable limb circumference despite weight reduction and caloric restriction, worsening of symptoms during the course of a day, visible vascular markings around fat deposits, and skin hypothermia [25]. Staging is based on structural skin changes and skin palpation (Table 1). In stage I, subjects have smooth skin with small nodules and reversible edema, and it is painful on occasion. In stage II, the skin is uneven and corrugated with walnut-sized nodules, reversible or irreversible edema, and thickened perilobar fascia with skin inflammation. In stage III, the skin is thickened and indurated, with disfiguring fat deposits, macronodular changes, and accompanying lymphedema. Stage IV lipedema acts in synergy with lymphedema [26] with large protruding portions of fat tissue on the legs and arms. In this stage, papillomatosis and cellulitis can also occur [27, 28].

**Table 1:** Stages of the lipedema

		***At the clinical investigation***
**Stage**	**I**	The skin appears smooth, the subdermis is enlarged, and there are small nodules in the hypertrophic subcutaneous adipose tissue layer. It may occasionally be painful and have a pebble-like texture due to loosening connective tissue fibrosis underneath.
	**II**	There are indentations on the skin caused by masses of varying sizes, ranging from the size of a pearl to an apple, that form in both the skin and adipose tissue. There are also palpable nodules and thickened perilobular fascia, which can contract and cause bands. The skin may appear inflamed due to advanced fibrotic changes, resulting in a “mattress pattern” where the skin is pulled down due to excess tissue.
	**III**	Increase in painful tissue that is more fibrotic in texture, with numerous large nodules located beneath the skin. The skin becomes thinner and loses its elasticity, allowing subcutaneous adipose tissue to grow excessively and fold over, which can impede fluid flow.
	**IV**	This condition is distinguished by the presence of lipolymphedema, which is the simultaneous occurrence of lymphedema and lipedema. It is marked by the presence of substantial amounts of fat tissue that hang over the legs or arms and by fat tissue extrusion on the legs.

With regard to the localization of the changes, lipedema has five types. Type 1 lipedema is characterized by fat tissue accumulation around the hips and buttocks, type 2 hips to knees, and type 3 hip to ankle. In more than 80% of the cases, arms are also involved, representing type 4 lipedema. Very rarely, lipedema is limited to arms exclusively or calves exclusively (type 5 lipedema) [1, 24, 27, 28]. According to Chakraborty, pain is reported by more than 90% of patients who report tissue pain and commonly affects the lower extremities, with affected areas increasing with the stage of lipedema [29]. Since lipedema is a diagnosis of exclusion, besides taking a detailed medical history and a physical examination, a complete biochemical analysis should be performed if lipedema is suspected, along with thyroid status and reproductive axis evaluation if there is rationale, excluding other possible causes of edema, and any disturbances found should be treated accordingly. Additional diagnostic tools that can be used to exclude other diseases or evaluate edema and the lymphatic system include ultrasonography, magnetic resonance imaging, computed tomography, indirect lymphography, and functional lymphatic scintigraphy, but they are not the first-line tests for lipedema diagnosis [25].

### Differential Diagnosis and Comorbidities

**Fig. 1:**
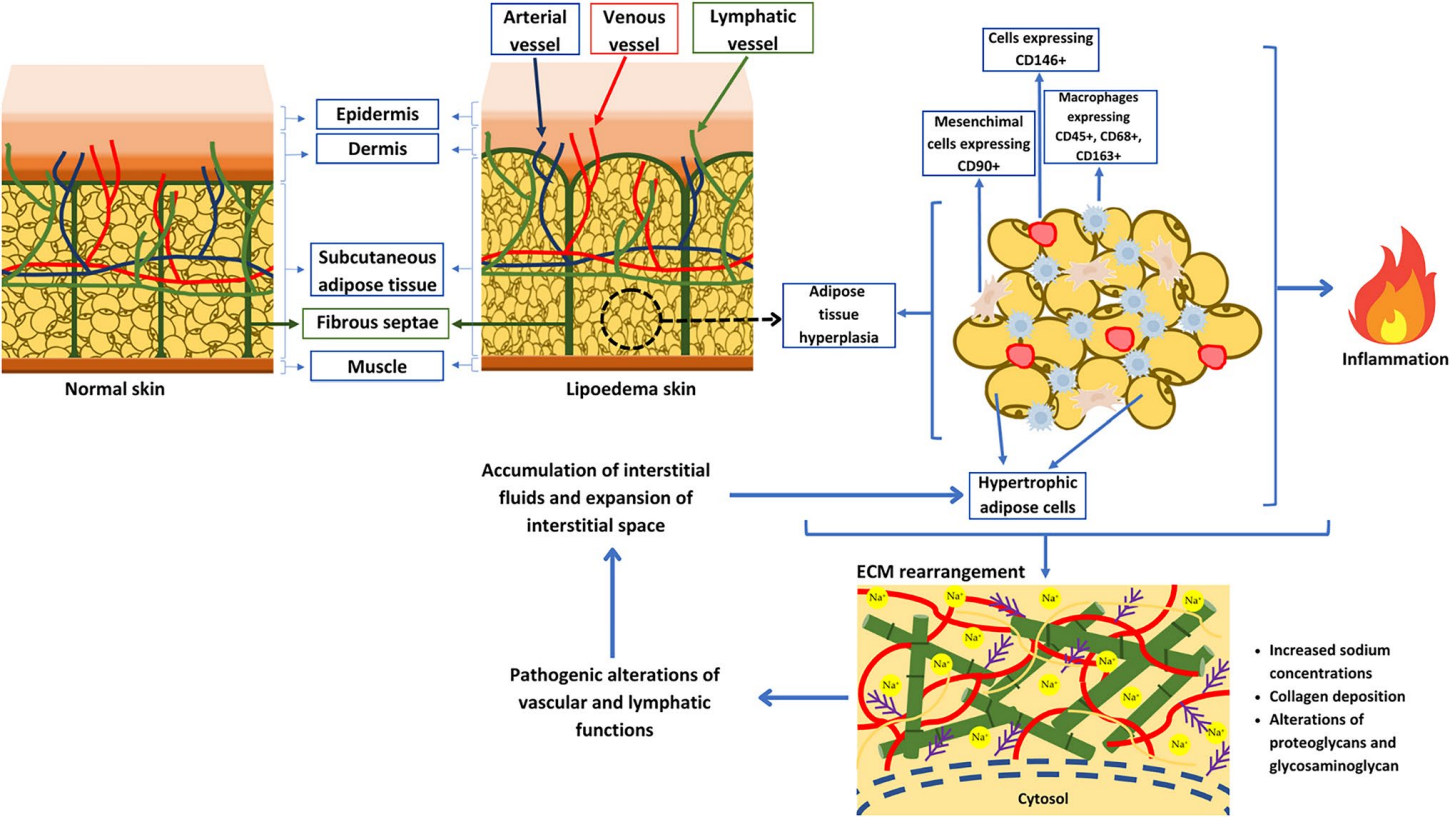
Macroscopic and microscopic features of subcutaneous tissue in lipedema. Lipedema is characterized by an increased accumulation of SAT in terms of augmented number of adipocytes (hyperplasia) due to the upregulation of mitotic clonal expansion genes. Such SAT expansion, accompanied by increased lipid accumulation within the adipocyte (hypertrophy), is responsible for (i) recruitment of immune cells and (ii) ECM rearrangement (increased Na+ concentration, collagen deposition, and alterations of proteoglycans and glycosaminoglycans). These two events result, on the one hand, in triggering inflammation and, on the other hand, in promoting pathogenic alterations of both vascular and lymphatic functions that, in turn, determine IF accumulation and IS expansion. The excessive IF surrounding adipocytes represents a source of nutrients, thus further promoting fat cell hypertrophy. Abbreviations: SAT, subcutaneous adipose tissue; ECM, extracellular matrix; IF, interstitial fluids; IS, interstitial space

Due to its elusiveness and underdiagnosis, it is important to differentiate the term lipedema from lymphedema, obesity, and lipohypertrophy since it is a distinct clinical entity, even though the pathogenesis is not yet fully understood (Fig. 1). Lipedema is always bilateral, with non-pitting edema unlike lymphedema, and a proper diagnostic algorithm can help rule out similar disorders such as lipodermatosclerosis, chronic venous insufficiency, obesity, and lymphedema [12]. Bilateral edema of the lower extremities can also be associated with other serious chronic conditions such as hypoalbuminemia, chronic kidney disease, congestive heart disease, pretibial myxedema, chronic venous insufficiency, and the use of certain medications like calcium channel blockers, gabapentin, and corticosteroids [30]. Lipodermatosclerosis consists of indurated skin, bilateral swelling of the shins, and erythematosus changes; however, in the acute phase, due to bilateral swelling, pain, and warmth, it can be like lipedema or cellulitis [31]. The clinical distinction between the lipid disorders remains challenging. High-resolution ultrasound diagnostic criteria are not successful at differentiating lipedema from other adipose tissue hypertrophy disorders [32]. The tissue water content in subjects with lipedema was also shown to be in the healthy control range [33, 34]. Other frequent comorbidities include cardiac disease (such as hypertension), thyroid disorders (as hypothyroidism), fibromyalgia, and type 2 diabetes mellitus [35]. However, according to Sanchez-De la Torre and colleagues, lipedema was associated with a low risk of diabetes (2%), despite an average BMI of 35.3 ± 1.7 kg/m^2^ [26]. Lymphedema does not cause lipedema, but subjects with obesity and lipedema are more prone to lymphedema and should opt for weight reduction [36].

### Assessment of Body Composition

As previously described, the diagnosis of lipedema is a challenging issue [24, 37], since physicians need to distinguish common gynoid obesity from this typical adipose tissue disorder [8, 38]. From a nutritional point of view, the most important aspect refers to a close follow-up during the nutritional treatment [24]. In this sense, German guidelines for the management of lipedema recommend monitoring anthropometric parameters (such as weight and circumferences) and derived indices and ratios (such as BMI and WHR) for both differential diagnosis and follow-up [24]. Nevertheless, such measurements present various limitations since they provide macroscopic information regarding body segments but do not allow an accurate study of body masses. For example, in subjects with lipedema, BMI needs to be contextualized in a more in-depth assessment since obesity is a typical feature of lipedema, but it is not an exclusive condition. Many studies on subjects with lipedema indeed reported a percentage of normal weight, although subjects with obesity were the majority [35, 39]. This suggests the importance of not relying solely on BMI calculations during a nutritional assessment of such subjects. In line with the above point, it should be noted that BMI does not provide information on general or local adiposity since body weight is used in the formula; thus, it does not distinguish fat mass from fat-free mass [40, 41]. A complete nutritional follow-up thus requests the combination of anthropometric measurements with other techniques licensed for the assessment of body composition, including the gold standards dual-energy x-ray absorptiometry (DXA) and BIA. Similarly, biochemical parameters should be monitored during the KD treatment, as reported in Table 3.

#### Circumferences

In subjects with lipedema, circumference measurement is a rapid, inexpensive, and repeatable method to monitor the course of treatments [42]. Studies on women with lipedema who underwent KD protocols reported significant reductions in upper limb, lower limb, waist, and hip circumferences [19, 20, 41]. It is worth noting, however, that circumference measurements provide indirect assessment of the subcutaneous tissues, but they do not allow discriminating the type of tissue is reducing [42]. Thus, in some cases, it is better to monitor indices obtained from them, including the WHR and Kuhnke disc methods [43, 44], the latter to measure limb volume. Although limb volume measurement has been described as appropriate in evaluating the course of treatments in subjects with lipedema, it presents the same limitation as other anthropometric measurements regarding the failure to distinguish the type of tissue being reduced [42].

#### Dual-Energy X-ray Absorptiometry and Body Impedance Analysis

DXA is currently licensed as the reference method for the assessment of body composition [45]. This technique has been used in clinical trials on subjects with lipedema not only because it is capable of accurately estimating body compartments in both whole body and single segments (trunk and limbs), but also because it allows discriminating lipedema from obesity [1, 34]. DXA, however, has intrinsic limitations related to high costs, technical expertise, and contraindications [45]. To deal with the main limitation related to the use of DXA, BIA is licensed as a reliable, noninvasive, and low-cost method to estimate body composition (fat mass and fat-free mass) and hydration status in terms of total body water, extra- and intracellular water, by measuring the electrical properties of body tissues [45]. The BIA-obtained estimation of body composition, however, depends on the predictive equation used and may be influenced by the hydration status. This suggests the importance of referring to BIA raw parameters in subjects with abnormalities in hydration [46]. This may be the case in subjects with lipedema who present higher values of extracellular water than controls [47], probably due to limb fat mass deposition causing an expansion of extracellular water compartment [48].

Among the others, phase angle (PhA) is conceivably the most important BIA-derived raw parameter since it derives from two direct values, resistance (R) and reactance (Xc) [PhA (°) = arc tangent (Xc/R) × (180/π)] [49]. In general, PhA reflects the integrity of cell membranes in a direct way. More specifically, low PhA values indicate decreases in cell integrity or cell death [49]. This allows for obtaining two main pieces of information on nutritional status. Firstly, PhA provides direct information on the water distribution between extra- and intracellular compartments [49]. Also, PhA provides information on the inflammatory status [49]. Studies have indeed reported a significant negative correlation between PhA values and phlogosis biomarker levels, including CRP [50–52]. Such an inverse association can be interpreted considering the tissue damage induced by chronic and unregulated inflammatory processes [53, 54], as typically occurs in cases of excess and dysfunction of adipose tissue with the well-known chronic low-grade inflammation [53, 55]. It appears clear, thus, the importance of using PhA in nutritional follow-up and in subjects with lipedema to monitor changes in inflammatory status. Interestingly, although PhA has been used to monitor inflammatory status variations in course of KD [52], limited data are available regarding changes in PhA values in subjects with lipedema treated with this dietary protocol. To our knowledge, only one study investigated in this sense, reporting significant increases in PhA values in a subject with lipedema during KD [20].

#### Segmental Bioimpedance Analysis

Advances in technology allowed for the development of novel devices for a BIA method aimed at estimating body composition in the different body districts, named segmental BIA (S-BIA), based on the concept that the body is composed of five cylinders, four limbs, and a trunk [56]. It is easy to understand thus the utility of S-BIA in subjects with lipedema in course of KD. Indeed, it has been reported that leg fat mass differed significantly between women with lipedema and women with obesity (without lipedema) [47], suggesting this method is also useful for differential diagnosis. Moreover, S-BIA allows monitoring body composition changes at limb level during nutritional treatments, as reported in subjects with lipedema who underwent KD, where higher reductions in leg fat mass were observed compared to non-KD [41]. The cost-effectiveness and safety, however, lead us to consider the use of S-BIA in clinical settings. Specific commercial S-BIA devices and software are currently available. However, a different approach for S-BIA might be speculated based on the measurement of the impedance of body segments. This can be accomplished using electrode positioning different from the canonical (wrist-to-ankle), including leg-to-leg, arm-to-arm, or five- or nine segment models, as previously described [57, 58]. A correct assessment of body masses is not possible. However, the obtained values of R and Xc might be used to mathematically calculate PhA, which, in this case, would refer to a single limb or segment, allowing for monitoring of the local inflammation in subjects with lipedema. Nevertheless, the lack of reference data and cut-offs makes it necessary to conduct studies in this sense.

Table 2 reports the main parameters and method to monitor and adopt during a KD.

**Table 2:** Main parameters to monitor in course of KD, according to the European guidelines by Muscogiuri et al. [17]

**Parameter/Method**	**Comments**
*Anthropometric measures*	
Body weight	It should be progressively and routinely monitored in course of KD, since a weight loss is expected. It does not provide information about an effective reduction of fat mass.
BMI	It should be progressively and routinely monitored in course of KD. It should be integrated with the assessment of body composition since it does not provide information on general or local adiposity.
Circumferences	They should be progressively and routinely monitored in course of KD. WHR obtained from waist and hip circumferences allows discriminating from gynoid (or iliofemoral or peripheral) and android (or visceral or central) adiposity. WHR > 0.85 is a valid proxy for central adiposity. Circumferences should be monitored at various reference point on arm and leg, including ankle (at the smallest point), calf (at the largest point), thigh (midway between patella and inguinal fold), and arm (midway between acromion process of shoulder and olecranon process of elbow). Limb circumference-derived method (Kuhnke disc method) allows measuring limb volume. With Kuhnke disc method limb volume is calculated measuring the circumferences at regular intervals of 4 cm from the ankle to the highest site of the inner thigh.
*Body composition and hydration status*	
DXA	It is the reference method for body composition assessment. It estimates accurately body compartments in both whole body and single segments and allows discriminating lipedema from obesity. It presents intrinsic limitations related to high costs, technical expertise, and contraindications.
BIA	It should be progressively and routinely monitored in course of KD. It allows body composition (fat mass and fat-free mass) and hydration status, in terms of total body water, extra- and intracellular water. The estimation of body composition depends on predictive equation and may be influenced by hydration status. Monitoring BIA raw parameters (i.e., PhA) is more reliable. PhA provides information on water distribution and inflammatory status. S-BIA provides an estimation of body composition in the different body districts, allowing differential diagnosis. S-BIA allows monitoring changes in limb fat mass in subjects with lipedema underwent KD.
*Blood parameters*	
Complete blood count with platelets	They should be progressively and routinely monitored in course of KD.
Electrolytes (Na, K, Mg, P)	They should be progressively and routinely monitored in course of KD. Ketone production causes increased urination, resulting in dehydration and electrolyte loss.
Renal and hepatic function parameters (creatinine, urea nitrogen, AST, ALT, γ-GT, albumin, bilirubin)	They should be progressively and routinely monitored in course of KD. A transient increase in uricemia can be detected under KD. KD is contraindicated in case of kidney and/or liver failure and chronic kidney disease.
Fasting glucose and insulin	They should be monitored at least at the beginning and end of KD. KD-induced fat loss improves glucose metabolism and insulin sensitivity.
Fasting lipids	They should be monitored at least at the beginning and end of KD. In general, KD is accompanied with decreases in triglyceride levels, unaffected HDL cholesterol levels, and a transient increase in LDL cholesterol level, mainly due to high fat intake.
Vitamin D and calcium	They should be monitored at least at the beginning and end of KD. An acid-ash protein rich diet may cause a calcium loss; however, calciuria seems not to increase the osteoporosis risk.
Thyroid function parameters (TSH, FT4)	They should be monitored at least at the beginning and end of KD.
β-Hydroxybutyrate (capillary blood or urine)	It should be monitored at least during the active stage of KD.
Complete urine analysis and microalbuminuria	They should be progressively and routinely monitored in course of KD.

*KD* ketogenic diet, *BMI* body mass index, *WHR* waist to hip ratio, *DXA* dual-energy x-ray absorptiometry, *BIA* body impedance analysis, *S-BIA* segmental body impedance analysis, *AST* aspartate aminotransferase, *ALT* alanine aminotransferase, *γ-GT* γ-glutamyl transferase, *TSH* thyroid-stimulating hormone, *FT4* tree thyroxine

### Treatment

Lipedema can be treated conservatively or surgically; however, both methods are aimed at relieving the symptoms, alleviating some of the difficulties these subjects face every day, and preventing further complications [12]. To date, there are no preventive measures, regardless of a known family history of lipedema. Conservative treatment should be accommodated individually according to the stage of disease, present comorbidities, and subjects’ overall well-being [14]. The two key aspects of management include psycho-social support such as psychotherapy, dietary counseling, and self-management education, along with physical therapy methods—lymph drainage, custom compression therapy, physiotherapy, exercise, and weight reduction [25]. Psychological treatment is very important due to the low efficacy of available treatments and the burden of psychosocial stress [26]. Exercise should be tailored and should include swimming, underwater exercises, and exercises that activate big muscle groups in the lower extremities, such as walking, cycling, and running [14, 37, 59]. Subjects with severe forms of lipedema can be also monitored in specialized hospital departments and units. A venous thromboembolism risk assessment and prophylaxis should be introduced where necessary [14]. Conservative treatment almost never improves the visual impairment of the affected extremities [25] and despite some pain and swelling relief, the reduction of tissue volume is considerably low (5–10%) [60–62]. Various diets have been explored as one of the modalities for lipedema; however, the data is still scarce. Mediterranean diet has been shown to be somewhat effective, while increasing intervals between meals and various anti-inflammatory diets have also been suggested [47]. However, to date, there is no randomized controlled trial that explores the effects of specific diets in the management of lipedema [25]. The efficacy of the surgical treatment with liposuction in lipedema treatment is promising, but it is still not sufficient to consider surgery as a first-line treatment. In stage IV, dermato-fibro-lipectomy could be performed since the classical liposuction would not have any reductive effect [63]. Lymph sparing liposuction was shown to be a safe procedure, reducing leg circumference and the frequency of other conservative treatment modalities, while subjects reported less pain, hematomas, and tenderness with a notable improvement in quality of life after surgery [64]. It is advised that the liposuction is performed sequentially, and subjects with significant amounts of body fat removal should be closely monitored for at least 12 h after the procedure [65–67].

### Expenditure in Health Care of Lipedema

Lipedema can have a significant impact on the global healthcare system and incur substantial costs for healthcare spending [68].

Firstly, the underdiagnosis and misdiagnosis of lipedema can result in delayed or inappropriate treatment, leading to increased healthcare utilization and costs. Lipedema is often mistaken for obesity or lymphedema, and patients may undergo unnecessary tests, consultations, and treatments before receiving an accurate diagnosis [12]. This not only places a burden on healthcare resources but also increases the financial strain on patients and the healthcare system.

Secondly, the management of lipedema requires a multidisciplinary approach involving various healthcare professionals, such as dermatologists, vascular specialists, nutritionists, and physiotherapists [69]. Coordinating the care of lipedema patients can be complex and may require specialized centers or clinics, further adding to the healthcare system’s workload and costs.

Thirdly, surgical interventions, such as liposuction, are often considered in severe cases of lipedema to alleviate symptoms and improve quality of life [64]. These procedures can be costly, particularly when combined with postoperative care and rehabilitation. However, the availability and accessibility of such surgical treatments may vary across different countries and healthcare systems, affecting the overall cost and quality of care provided to lipedema patients.

Lastly, the psychosocial impact of lipedema, including decreased self-esteem and impaired quality of life, can result in additional healthcare utilization and costs [70]. Patients may seek psychological support, counseling, or specialized therapies to address the emotional and mental health aspects associated with the condition.

Overall, the impact of lipedema on the global healthcare system is multifaceted. It includes the costs associated with misdiagnosis, specialized care, surgical interventions, and the management of associated psychosocial issues. Improving awareness, early diagnosis, and access to appropriate care are essential in reducing the burden on healthcare systems and optimizing resources for the effective management of lipedema.

Solely recently, on January 1, 2022, lipedema was included in the International Classification of Diseases (ICD-11) of the World Health Organization as a separate clinical entity in the category “Certain noninflammatory disorders of subcutaneous fat,” code EF02.2 [71]. According to the ICD-11 description, “Lipedema is characterized by non-pitting diffuse ‘fatty’ swelling, usually confined to the legs, thighs, hips, and upper arms. It may be confused with lymphoedema” [70].

## Lipedema-Obesity-Inflammation: A Vicious Cycle

**Fig. 2:**
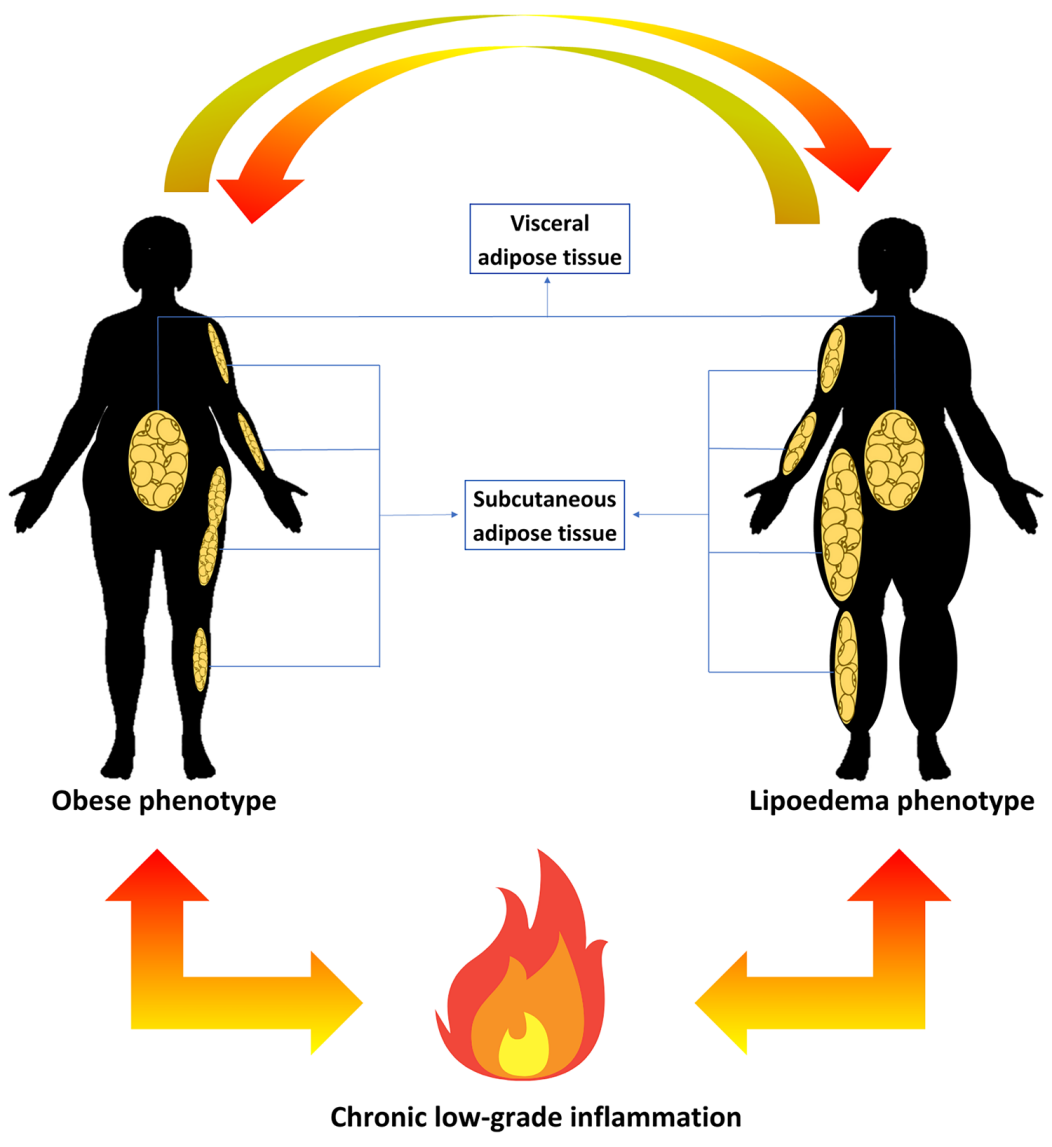
The obesity-lipedema-inflammation vicious cycle. Obesity is a typical lipedema feature, but it is not an exclusive condition. These two phenotypes, however, are strongly related, with a bi-directional relationship. Both conditions are characterized by fat accumulation at the visceral but also subcutaneous level, especially in the upper and lower limbs, in the higher entity (grater SAT expansion) in lipedema. Obesity and lipedema can be mutually converted into each other, and both contribute to trigger and/or exacerbate a flogosis status, contributing to the development of a chronic low-grade inflammation condition. Inflammation, in turn, is responsible for impairment in vascular and lymphatic function, insulin resistance, empowerment of androgenic activity, and the development of metabolic disorders that contribute to further accumulation of adipose tissue, promoting hypertrophic and hyperplastic processes, thus perpetuating the cycle. Abbreviation: SAT, subcutaneous adipose tissue

Obesity, characterized by hypertrophy and/or hyperplasia of adipose tissue, leads to a chronic inflammatory state [72], which acts as a fundamental underlying mechanism for the development of obesity-related diseases [73]. The chronic inflammatory state of obesity is characterized by increased levels of inflammatory mediators, which can induce cell damage and cell death through apoptosis or necrosis [74]. As in obesity, recent evidence points to the presence of a chronic inflammatory state in lipedema. Moreover, it appears that this chronic inflammatory state in lipedema is independent of the co-presence of obesity (Fig. 2).

Adipose tissue is composed of various cell types, including adipocytes, immune cells, blood cells, and endothelial cells [75]. The growth and function of white adipose tissue are regulated by communication between these cells, with a central role for the vascular endothelium [76]. The endothelium regulates the delivery of nutrients to adipocytes through tightly arranged capillaries [76]. When adipose tissue reaches its maximum growth rate, it loses the ability to store lipids, and can lead to lipid leakage and metabolic deterioration [77]. In this regard, lipedema is a condition where adipose tissue is pathologically altered, including impairment of the adipose vasculature, interstitial fluid and lymphatic system, extracellular matrix remodeling, and collagen deposition, leading to fibrosis and edema [26]. Subjects with lipedema showed hypertrophic and hyperplastic adipocytes, increased intercellular fibrosis, elevated macrophage levels, and morphological differences compared to healthy control tissue [78]. Lipedema leads to structural and functional reprogramming [78], including increased lipid release [79], tissue inflammation, and excessive fluid accumulation [80]. Adipose hypertrophy in lipedema causes swelling in a symmetrical form without interstitial edema, but excessive fluid accumulation can occur in progressed lipedema due to various factors such as increased capillary pressure and inadequate lymphatic outflow [80]. The increased interstitial fluid in lipedema allows for the palpation of individual fat lobules as nodules [81], and slow blood and lymphatic flow can lead to inflammatory and fibrotic lesions, chronic pain, and palpation [82]. The lymphatic system is not compromised in the early stages and can cope with increased interstitial fluid, but subjects with lipedema may develop secondary lymphedema (lipolymphedema) if excessive adipose tissue depots compromise the lymphatic system.

The lymphatic vasculature in lipedema shows increased permeability with a larger interstitial space [83]. Several novel potential biomarkers for lipedema in the last few years have been described [13]. Platelet factor 4 has shown promising results as a biomarker for lymphatic diseases, both in animal models and human studies [84]. Tissue sodium has also been considered potentially relevant since subjects with lipedema show reduced sodium clearance from interstitial spaces, which could be associated with adipose accumulation [85]. Lipedema tissue exhibits inflammation leading to tissue fibrosis in the extracellular matrix and the formation of palpable fibrotic nodule-like structures within the skin [82]. Aberrant elastic fibers are seen in skin lesions, and pearl-sized nodules close to lymph nodes are features of lipedema in subcutaneous adipose tissue, representing tenderness to palpation [79]. Hyperechoic masses in lipedema can indicate a leaky vessel, a bruise, or inflammation around a vessel [80]. It therefore appears that the chronic state of inflammation in lipedema is not related to the presence of obesity but to the condition itself. In this regard, Al-Ghadban and colleagues conducted a study in 49 women (40 with lipedema and 19 controls) and divided them according to the presence or absence of obesity (respectively, a BMI above or below 30.0 kg/m^2^) [86]. They showed that a set of alterations (hypertrophic adipocytes, increased numbers of macrophages and blood vessels, dilated capillaries in the thigh tissue) that have already been highlighted in obesity as triggering inflammation were, however, also present in women with lipedema and without obesity. The authors concluded that this suggests the presence of a chronic inflammatory state in women with lipedema, independent of obesity [86]. Moreover, Priglinger et al. characterized the cells of the stromal vascular fraction of 30 subjects with lipedema compared to 22 healthy subjects [87]. Interestingly, their results also revealed a greater increase in Let-7c miRNAs in subjects with lipedema than in controls, and that Let-7c is known to promote the polarization of macrophages towards the M1 phenotype. M1 macrophages are pro-inflammatory and produce cytokines such as IL-1β, IL-6, IL-12, IL-23, and tumor necrosis factor (TNF)-α. This would also argue for a chronic inflammatory state in lipedema [87]. The main mechanisms and histological alterations underlying lipedema development and progression are schematically represented in Fig. 1.

Thus, lipedema, obesity, and inflammation are interconnected in a vicious cycle (Fig. 2). Lipedema can lead to obesity, which can further exacerbate the inflammatory state of the body [88]. Inflammation, in turn, can contribute to the development and progression of lipedema. The accumulation of excess adipose tissue in lipedema can lead to chronic inflammation, which can impair lymphatic function and cause further accumulation of adipose tissue [13]. The chronic inflammation associated with obesity can also lead to insulin resistance, which can contribute to the development of metabolic disorders [55]. The resulting metabolic dysfunction can further exacerbate the inflammatory state, which perpetuates the cycle. Therefore, breaking this cycle requires a multifaceted approach that includes weight management, anti-inflammatory interventions, and treatment for underlying medical conditions.

## Nutritional Treatment for Lipedema: Ketogenic Diet

To effectively counteract the clinical manifestations and symptoms of lipedema, diet and exercise adjustments are utilized. According to Kruppa and colleagues, there is no specific evidence-based diet for subjects with lipedema [25]. To date, no effective nutritional treatment for subjects with lipedema has been reported. Current dietary approaches are generally based on empirical data and aim to reduce body weight through a low-calorie diet, inhibit systemic inflammation with antioxidant and anti-inflammatory components, and reduce water retention [25, 89]. However, it has been shown that subjects with lipedema are highly resistant to conventional diet and exercise interventions [90]. Indeed, any weight loss in subjects with lipedema resulting from conventional diet and exercise approaches will inevitably only occur in the upper body, resulting in increased asymmetry and further body dysmorphia [90]. Moreover, in subjects with insulin resistance, increased lipolysis and altered lipogenesis in adipose tissue lead to the release of cytokines and lipid metabolites, perpetuating insulin resistance and a low-grade chronic inflammation state [55]. Consequently, the few approaches studied in the literature mainly concern low-carbohydrate diets with the effect of reducing inflammation and insulin levels and thus adipogenesis [90].

Most recently, subjects’ experiences seem to indicate that KD may influence weight and symptom management in lipedema [19, 20]. Cannataro and colleagues described a 32-year-old female clinical case with a diagnosis of lipedema types IV and V, stages II–III; she complained of widespread pain, particularly in her lower limbs, heaviness, and difficulty performing various movements [20]. She refused any kind of treatment unless it was nutritional. This subject was fed a KD with a calorie deficit of approximately 250 kcal. The program provided an average intake of 1300 kcal, divided into 30% protein, 66% fat, and 4% carbohydrates, for 6 months. After the ketogenic phase, a low carbohydrate and low-calorie diet was followed. The results in terms of weight were striking: the subject lost 41 kg, with a change of around 20% in fat mass, as assessed by bioelectrical impedance analysis (BIA). Moreover, body circumferences showed a decrease in all districts: 37.5 cm less on the hips and 23.9 cm less on the waist; however, it should be emphasized that there were also significant decreases in body areas typically affected by lipedema, such as arms (−10.5 cm left, −11.5 cm right), forearms (−6.5 cm both), knees (−8.5 cm both), calves (−9 cm left, −8.5 cm right), and ankles (−2.5 cm left, −3 cm right). In addition, biochemical–clinical parameters showed that renal and hepatic function were not negatively affected; on the other hand, basal insulin decreased significantly (29.3 mIU/L vs. 14 mIU/L after the ketogenic phase), moderating the insulin resistance present in the initial state, as evidenced by the homeostatic model assessment for insulin resistance (HoMA-IR) index (7.16 vs. 3.28). C-reactive protein (CRP) levels were decreased, even if not elevated from the beginning, which could be a sign of less inflammation (0.6 mg/dL vs. 0.2 mg/dL). Lastly, in the four questionnaires for the assessment of the quality of life (QoL) (RAND-36, WOMAC, SQS, and VAS) administered during the period, a clear improvement was shown, even in daily actions, in the quality of sleep, and in the perception of the pathology as a more manageable, albeit limiting, condition.

Sorlie and colleagues aimed to evaluate, in a pilot study (LIPO DIET), the impact of a low-carbohydrate, high-fat (LCHF) eucaloric diet on pain and QoL in subjects with lipedema [19]. Nine women with a diagnosis of lipedema (BMI 36.7 ± 4.5 kg/m^2^, aged 46.9 ± 7 years), including all types and stages of lipedema affecting the legs, were enrolled and underwent a 7-week LCHF diet (fat 70–75%, carbohydrate 5–10%, and protein 20% of energy intake, respectively), followed by a 6-week diet according to Nordic nutrition recommendations. Pain (visual analogue scale) and QoL (limb lymphedema questionnaire), weight, and body composition were measured at baseline, week 7, and week 13. At the end of the study, the LCHF diet induced a significant weight loss (−4.5 ± 2.4%, *p* < 0.001), which was maintained at week 13 (−4.0 ± 2.4%, *p* < 0.001). No significant change in body weight was seen from week 7 to week 13 (*p* = 0.430). There was a significant decrease in waist (98.3 ± 2.7 vs. 94.0 ± 2.7 cm, *p* < 0.001) and hip (125.2 ± 1.6 vs. 123.0 ± 1.6 cm, *p* = 0.010) circumferences from baseline to week 7, and this was maintained at week 13. There was a significant decrease in calf circumference (48.0 ± 3.8 vs. 47.0 ± 3.8 cm, *p* = 0.030) from baseline to week 7, but no significant change in thigh circumference (67.0 ± 3.0 vs. 65.0 ± 3.0 cm, *p* = 0.200) during the same period. Moreover, the LCHF diet induced a significant reduction in pain from baseline to week 7 (4.6 ± 0.69 vs. 2.3 ± 0.69 cm, *p* = 0.018). Perceived pain returned to baseline levels at week 13 (4.2 ± 0.69 cm, *p* = 0.69). A significant increase in overall QoL was found between baseline and week 7 (*p* = 0.050) and week 13 (*p* = 0.050), respectively.

From the few studies available to date, KD is effective not only for rapid weight loss but also in improving pain symptoms and quality of life in subjects with lipedema (Table 3).

**Table 3:** Studies on the ketogenic diet in subjects with lipedema

**Reference (year)**	**Aim**	**Population**	**BMI** **kg/m**^**2**^	**Diet protocol**	**Main results**
Sørlie et al. (2022) [19]	To assess the impact of an eucaloric low carbohydrate, high fat diet on pain and QoL in subjects with lipedema	9 women with lipedema (aged 46.9 ± 7 years). Type and stage not reported	36.7 ± 4.5 kg/m^2^	Eucaloric low carbohydrate, high fat diet (fat 70–75%, carbohydrates 5–10%, proteins 20%) for 7 weeks thereafter 6 weeks of a diet following the Nordic nutrition recommendations	The low carbohydrate, high fat diet induced a significant weight loss (−4.5 ± 2.4%, *p* < 0.001) and reduction in pain (−2.3 ± 0.4 cm, *p* = 0.020). Weight loss was maintained between week 7 and 13, but pain returned to baseline levels at week 13. A significant increase in general QoL was found between baseline and week 7 (1.0 (*p* = 0.050) and 13 (*p* = 0.050), respectively
Cannataro et al. (2021) [20]	To confirm the hypothesis on the efficacy and safety of the ketogenic diet in subjects with lipedema, even when maintained for a long time	A 32-year-old woman diagnosed with lipedema type IV and V, stage II–III	Not reported	Ketogenic diet (fat 66%, 30% proteins, 4% carbohydrates) for 24 months (6 months followed optimally, 18 not always optimally)	The woman achieved a significant weight loss (−41 kg), with a net decrease in body circumferences, and also reported an improvement in pain, and in the overall quality of life

*BMI* body mass index, *QoL*, quality of life

## Differences Between a Ketogenic Diet and a Very Low-Calorie Ketogenic Diet

KDs are high-fat diets characterized by a carbohydrate restriction (30–50 g *per* day) [91]. The drastic reduction in the content of exogenous carbohydrates from the diet drives the body into physiological ketosis, i.e., into a metabolic state characterized by an increase in the concentration of ketone bodies in the blood. Ketone bodies are three products of hepatic ketogenesis, acetoacetate (AcAc), acetone (Ac), and β-hydroxybutyrate (βHB), although it is not defined as a ketone by IUPAC nomenclature. There are various KD protocols that differ from each other based on calories, percentages of different macronutrients, and the achievable ketogenic ratio (KR). The term KR refers to the ratio (R) between the amount of lipids in grams in the diet protocol and the amount of protein and carbohydrates. The main KD therapies that are used to treat obesity exploit nutritional ketosis, induced not only by the low-carbohydrate ratio but also by calorie reduction, to achieve a rapid and steady loss of fat mass while preserving lean mass. They are low calorie ketogenic diet (LCKD) and VLCKD. Recently, VLCKD was shown to determine significant weight loss along with improvement of glycemic control in subjects with obesity and type 2 diabetes mellitus [92–94]. According to the Position Statement of the Italian Endocrinology Society, the VLCKD protocol is characterized by a low daily calorie diet of 700–800 kcal/day with carbohydrate restriction of 30–50 g/day (≃13% of total energy intake), a 30–40 g/day (≃44%) increase in fats, and about 1.2–1.4 g/day proteins *per* kg body weight (≃43%) [92, 95]. Although often mistakenly considered a high-protein diet, VLCKD keeps daily protein intake at around 1.2 to 1.5 g/kg of ideal body weight. In addition, VLCKD is based on high biological protein from non-animal and/or animal protein sources, such as peas, eggs, soy, and whey protein [92]. Subjects with lipedema generally have a BMI greater than 25.0 kg/m^2^. Given the significant deposition of subcutaneous adipose tissue and the necessity to reduce it promptly in order to alleviate pain symptoms, inflammation, and edema, VLCKD would seem to be a more targeted and suitable dietary treatment, as a low-calorie and fat proportion is present.

## The Very Low-Calorie Ketogenic Diet (VLCKD) Protocol

The VLCKD protocol is divided into several steps [17, 92]. The initial VLCKD step, also called the active phase, is characterized by a very low-calorie diet (650–700 kcal/day), which is low in carbohydrates (< 30 g/day from vegetables) and fat (only 20 g/day, also from olive oil). The amount of high-biological-value protein varied between 1.2 and 1.5 g *per* kg of ideal body weight in order to preserve lean mass and meet the minimum daily body requirement. Due to the drastic reduction in carbohydrate content, the initial phase is also called the “active phase,” in which a controlled nutritional ketosis is maintained. The active phase is commonly divided into two phases: during phase 1, subjects eat high-biological-value protein meals four to five times a day, depending on gender, body weight, and physical activity, together with low glycemic index vegetables. The daily calorie intake is 600–700 kcal/day; during phase 2, however, one of the protein portions is replaced by natural protein such as meat/egg/fish at lunch or dinner. The daily calorie intake is 800 kcal/day [17, 92]. As this is a very low-calorie nutritional scheme, it is recommended to supplement subjects with micronutrients (vitamins such as B-complex vitamins, vitamin C and E and minerals including potassium, sodium, magnesium, calcium, and omega-3 fatty acids) according to international recommendations [96]. The initial step of VLCKD, in which nutritional ketosis is sustained, lasts until the subject loses most of the target weight, about 80% [17, 92]. Therefore, the active phases are time-varying depending on the individual and the weight loss target. According to the Position Statement of the Italian Society of Endocrinology, the active phase generally lasts between 8 and 12 weeks [92]. In phase 3, a second portion of natural low-fat protein preparation replaces the second portion of organic protein preparation and only 2–3 meal replacements are maintained. After these phases of the active phase, the subject switches to a low-calorie carbohydrate diet (LCCD). At this point, subjects gradually reintroduce different food groups, eliminating meal replacements. In addition, carbohydrates are gradually reintroduced, starting with the foods with the lowest glycemic index (such as dairy products and fruit). Typically, low-carbohydrate diets prescribe 60–130 g of carbohydrates *per* day (≤ 20–45% of daily energy intake). The daily calorie intake in phase 3 is between 1000 and 1200 kcal/day. In phase 4, a low-calorie diet (LCD) is followed, with a calorie intake of 1300 to 1400 kcal; pulses and whole-meal bread are reintroduced, and only 1–2 replacement meals are maintained. In phase 5, a hypocaloric Mediterranean diet (HMD) is maintained, following a Mediterranean diet with a caloric intake between 1500 and 1700 kcal, introducing cereals and pseudocereals with a low glycemic index; no replacement meals are maintained. This last step, through the acquisition of correct eating habits, is crucial for maintaining long-term results [17, 92]. As reported, it is essential for the subject with obesity to lose at least 15% of their body weight and maintain this loss in the long term to reduce cardiometabolic risks.

In this regard, it is important to highlight that both VLCKD and Mediterranean diet have their own advantages and disadvantages when it comes to lipedema. As previously mentioned, VLCKD can contribute to weight loss and ameliorate inflammation. However, it is associated with potential nutrient deficiencies, limited food choices, and a lack of long-term research [97]. On the other hand, the Mediterranean diet provides a nutrient-rich approach that promotes heart health and also possesses anti-inflammatory properties [15]. Nevertheless, its effects on weight loss and lipedema may be limited [98]. However, by combining these two dietary approaches, the beneficial effects for subjects with lipedema may be enhanced. A recent study demonstrated that high adherence to the Mediterranean diet prior to starting VLCKD improved the efficacy of the latter in terms of weight loss and improvements in body composition in women with overweight/obesity [99]. The presence of bioactive compounds in Mediterranean diet that favor a more effective metabolic setup for ketosis was suggested as the reason for these positive outcomes [99]. Furthermore, it should be noted that a Mediterranean-style nutritional approach also represents the maintenance phase of the VLCKD protocol [17, 92]. Therefore, it can be hypothesized that combining both dietary approaches may maximize the beneficial effects of the intervention in subjects with lipedema.

## Conclusions

**Fig. 3:**
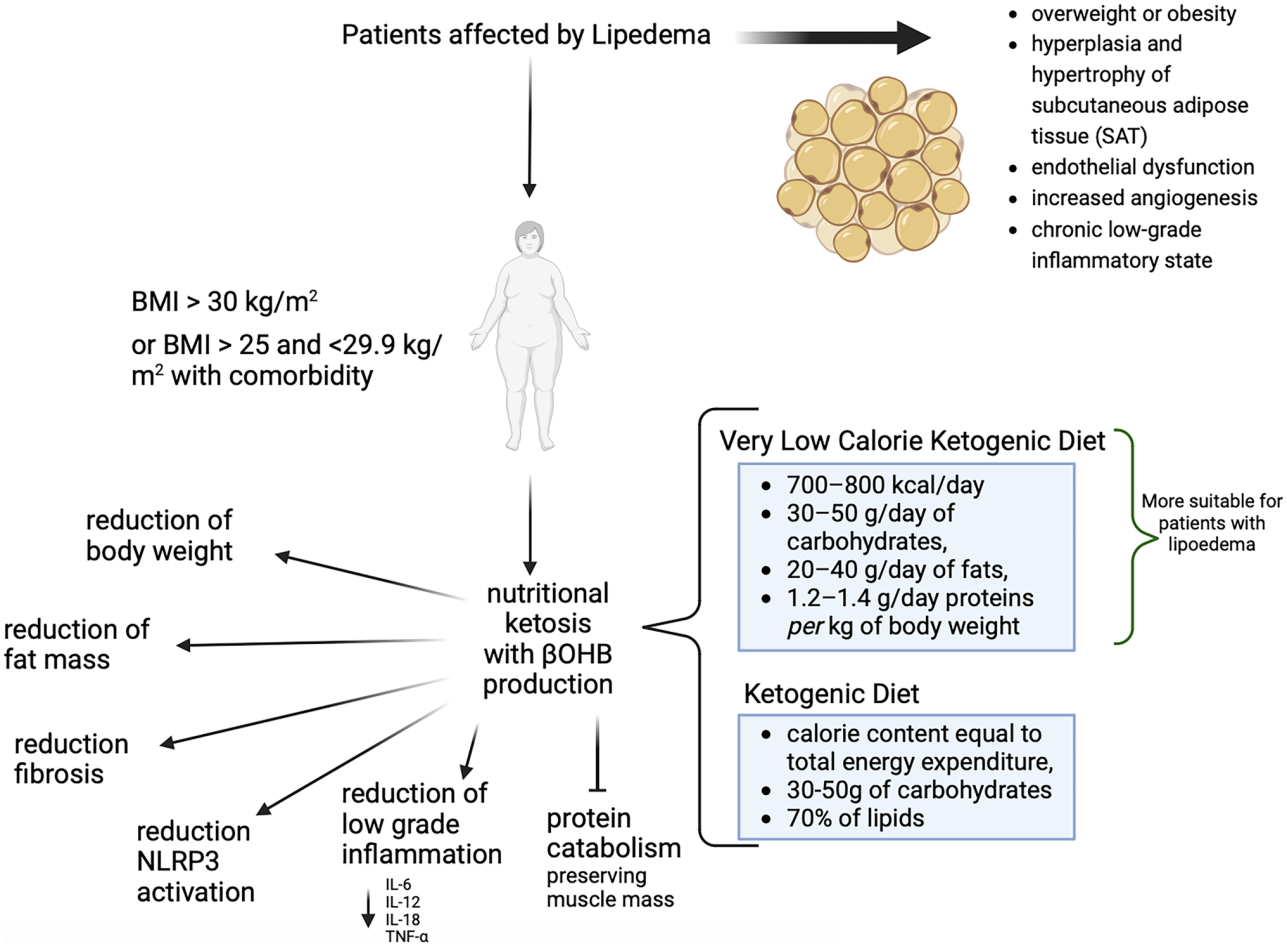
Very low-calorie ketogenic diet (VLCKD) for subjects with lipedema. Lipedema is often misdiagnosed as obesity or lymphedema, but its adipose tissue accumulation is resistant to lifestyle interventions. Nutritional strategies such as the VLCKD have shown potential for reducing inflammation, which is a significant contributor to lipedema’s pathogenesis. However, the evidence for the efficacy and safety of VLCKD in the treatment of lipedema is currently limited, and more research is needed to establish its efficacy

In conclusion, lipedema is a chronic and debilitating disease that predominantly affects females, and its diagnosis and etiology remain unclear. Hormonal alterations and genetic components, such as the AKR1C1 gene mutation, are thought to play a role in its pathophysiology. Lipedema is often misdiagnosed as obesity or lymphedema, but its adipose tissue accumulation is resistant to lifestyle interventions. Nutritional strategies such as VLCKD have shown potential for reducing inflammation, which is a significant contributor to lipedema’s pathogenesis (Fig. 3). However, the evidence for the efficacy and safety of VLCKD in the treatment of lipedema is currently limited, and more research is needed to establish its efficacy.
